# Unraveling Monkeypox: An Emerging Threat in Global Health

**DOI:** 10.7759/cureus.43961

**Published:** 2023-08-23

**Authors:** Abdullah Shehryar, Raghu Halappa Nagaraj, Fnu Kanwal, Shivani M Reddy, Han Grezenko, Yogesh Raut, Muhammad U Fareed, Abdur Rehman, Defne Şahin, Danyal Bakht, Palash Ramteke

**Affiliations:** 1 Internal Medicine, Allama Iqbal Medical College, Lahore, PAK; 2 Surgery, Avalon University School of Medicine, Willemstad, CUW; 3 Medicine, Chandka Medical College, Larkana, PAK; 4 Student, Chalmeda Anand Rao Institute of Medical Sciences, Karimnagar, IND; 5 Translational Neuroscience, Barrow Neurological Institute, Phoenix, USA; 6 Medicine, Narendra Kumar Prasadrao (NKP) Salve Institute of Medical Sciences, Nagpur, IND; 7 Department of Surgery, Nishtar Medical University, Multan, PAK; 8 Surgery, Mayo Hospital, Lahore, PAK; 9 Internal Medicine, Psychiatry, Ege University Hospital, İzmir, TUR; 10 Medicine and Surgery, Mayo Hospital, Lahore, PAK

**Keywords:** historical progression, global health issue, central and west africa, viral zoonotic ailment, monkeypox virus

## Abstract

Monkeypox, a viral zoonotic ailment originating in the Central and West African regions, has escalated into a global health issue of growing concern. The current analysis offers an exhaustive examination of monkeypox, emphasizing its historical progression, etiology, epidemiological patterns, pathophysiological mechanisms, clinical manifestations, diagnostic methodologies, treatment modalities, and preventive strategies. The worldwide discontinuation of smallpox vaccination has contributed to an increased incidence of monkeypox, driven by the expansion of vulnerable host populations. Significant strides in diagnostic procedures, prospective antiviral treatments, and vaccine development exhibit potential in managing this affliction, yet obstacles remain in terms of disease control, prevention, and treatment. Additionally, the international propagation of monkeypox underscores the need for robust public health initiatives and the significant role played by global health institutions in disease containment. Prospective research endeavors should strive to enhance our comprehension of the natural reservoirs of monkeypox and its transmission dynamics, evaluate sustained immune responses to novel vaccines, and investigate the potential impact of One Health strategies. This analysis underscores the pressing necessity for increased research and synchronized global efforts to tackle this emergent infectious malady.

## Introduction and background

Monkeypox, a viral zoonotic ailment, is emerging as a mounting concern for global health [1]. Its roots trace to Central and West Africa and it was initially identified in 1958 during outbreaks of a pox-like condition in research monkeys [2]. The inaugural human case was subsequently reported in 1970 in the Democratic Republic of Congo [3].

Monkeypox is the product of the monkeypox virus, part of the orthopoxvirus genus, which also includes variola virus (smallpox's causative agent), vaccinia virus (utilized in the smallpox vaccine), and cowpox virus [4]. The disease manifests in humans in a manner akin to but less severe than smallpox. Nonetheless, it has been linked to considerable morbidity and mortality, especially in areas with low smallpox vaccination coverage [5].

The successful global eradication of smallpox in 1980 and the subsequent termination of routine smallpox vaccination have led to a growing pool of unvaccinated individuals, thus paving the way for a susceptible host reservoir for the monkeypox virus [6]. Over the previous two decades, the prevalence of human monkeypox has seen a considerable increase, particularly in Central and West Africa [7]. The disease has also emerged in the United States, the United Kingdom, Israel, and Singapore, illustrating its potential for worldwide spread [8].

This review is designed to offer a thorough examination of monkeypox as an escalating threat to global health. We aim to dissect various aspects of the disease, encompassing its history, etiology, epidemiology, pathophysiology, clinical manifestations, diagnostic procedures, treatment options, and preventive measures. Additionally, we will address the ongoing challenges and debates concerning monkeypox management and highlight recent advancements and future outlooks in research. Through this review, our objective is to enlighten and instruct clinicians, researchers, and policymakers about the escalating peril of monkeypox and the necessity for robust public health interventions to curb its proliferation [9].

## Review

Disease overview

Historical Account and Discovery of Monkeypox

The term "monkeypox" was first coined in 1958 following outbreaks of a disease resembling pox among monkeys housed in research laboratories in Denmark and the United States [10]. These incidents led to the discovery of an unknown virus, which was initially mistaken for a variant of smallpox [11]. However, further analysis clarified that this was a novel virus, and it was subsequently named the "monkeypox virus" due to its initial identification in monkeys [11].

The first recorded case of monkeypox in humans was reported in 1970 in the Democratic Republic of Congo during a heightened campaign to eliminate smallpox [12]. Clinically, the symptoms of monkeypox in humans bore a resemblance to those of smallpox, though they were typically less severe [13]. Notably, despite their similarities, there was a clear distinction between the two diseases: vaccination against smallpox did not provide complete protection against monkeypox infection [14].

Etiology and Taxonomic Classification of Monkeypox Virus

The monkeypox virus is a member of the orthopoxvirus genus, which falls under the broader *Poxviridae *family [15]. This genus also includes other notable viruses such as the variola virus, responsible for smallpox; the vaccinia virus, which is utilized in the smallpox vaccine; and the cowpox virus [15]. It's important to note that while these viruses share a genus, each one causes a distinct disease in humans.

Characteristically, like its counterparts in the poxvirus group, the monkeypox virus possesses a large and intricate structure, containing a linear, double-stranded DNA genome [16]. Demonstrating zoonotic characteristics, the virus can be transmitted from animals to humans. Rodents and monkeys are identified as the primary animal reservoirs for this virus [17].

Furthermore, the monkeypox virus is divided into two main clades: West African and Central African. These clades exhibit variances in their virulence levels when infecting humans. Notably, infections stemming from the Central African clade tend to result in more severe manifestations of the disease [18].

Epidemiology

Prevalence and Geographic Distribution

Monkeypox is primarily observed in remote regions of Central and West Africa, in proximity to tropical rainforests [7]. A surge in the number of reported monkeypox cases in humans has been seen in these areas in recent years [19]. For instance, a significant escalation in human monkeypox incidence has been reported in the Democratic Republic of Congo, particularly following the cessation of routine smallpox vaccination [20].

Beyond Africa, occasional instances of monkeypox have been noted globally, attesting to the disease's potential for worldwide dispersion. For example, the United States witnessed a monkeypox outbreak in 2003, traced back to imported pet rodents from Ghana [21]. More recently, the United Kingdom reported its inaugural two monkeypox cases in 2018, imported from Nigeria [8].

Modes of Transmission

The monkeypox virus primarily spreads to humans when they are bitten by an infected animal or come into direct contact with the animal's blood, bodily fluids, or open sores [22]. Notably, rodents like squirrels and rats, as well as monkeys, are considered the main animal carriers of the monkeypox virus [17].

Additionally, the virus can be transmitted from one human to another. This human-to-human transmission typically occurs when an individual is in close proximity to or directly touches the skin lesions or bodily fluids of an infected person. Moreover, the virus can propagate via respiratory droplets, especially during prolonged face-to-face interactions or close contact with an infected individual, often within household settings [23].

Risk Factors

Several risk factors can increase the likelihood of contracting monkeypox. Foremost among these is close contact with animals or humans who are infected [20]. In certain areas of Central and West Africa, traditional practices such as hunting and consuming rodents and primates further amplify the risk of transmission from animals to humans [24]. Additionally, healthcare professionals attending to monkeypox patients, as well as family members living in the same household as an infected person, face a heightened risk of human-to-human transmission [13].

Pathophysiology

Mechanisms of Disease

Once the monkeypox virus enters the body, it primarily invades the regional lymph nodes, where it begins to replicate [25]. Following this, the virus spreads throughout the body via the bloodstream in two distinct phases. The first phase, known as primary viremia, presents as a mild, non-specific febrile illness [22].

The second phase, or secondary viremia, specifically targets certain cells within the body, including skin cells. This leads to the characteristic rash and pox lesions that are associated with monkeypox [26]. Approximately one week after the onset of initial symptoms, these skin lesions emerge and progress through various stages, starting from macules, then to papules, vesicles, pustules, and finally, scabs [27].

It's important to highlight that the monkeypox virus has developed mechanisms to evade the host's immune response. This evasion allows the virus to continue its replication and systemic spread within the host body [16].

Progression of Disease and Stages

Monkeypox is a viral disease whose clinical progression can be understood through three distinct phases: the invasion phase, the eruptive phase, and the convalescent phase [28].

The invasion phase, which typically lasts between 1 to 3 days, marks the onset of the disease. During this period, affected individuals often experience a range of symptoms. Fever, headache, muscle pain, backache, and swollen lymph nodes are common, often accompanied by chills and a general sense of fatigue. These initial symptoms serve as the body's immediate response to the viral invasion and can be quite debilitating for the patient [29].

Following the invasion phase, patients enter the eruptive phase. This is most notably characterized by the emergence of a distinctive rash. Initially appearing on the face, this rash gradually spreads to other parts of the body. As the days progress, these skin lesions undergo a series of transformations. They begin as flat spots, or macules, which then elevate into papules. These papules subsequently fill with fluid, becoming vesicles, and later turn into pustules. Over time, these pustules dry out, forming crusts or scabs. The entire process, from the appearance of macules to the formation of scabs, provides a visual representation of the body's battle against the Monkeypox virus [7].

The final stage in the clinical progression of monkeypox is the convalescent phase. This phase signifies the body's recovery from the illness. It begins as the crusts from the lesions start to fall off and can extend for several weeks. While the body's natural defenses typically manage to overcome the virus, making the disease self-limiting, complications can arise. Individuals with compromised immune systems are especially at risk. For these individuals, monkeypox can escalate in severity and, in rare cases, can even prove lethal [20].

Clinical manifestations

Clinical Characteristics

The clinical presentation of monkeypox bears similarities to, but is generally less severe than, smallpox. Following an incubation period of 7-14 days, the disease onset is marked by fever, headache, myalgia, backache, lymphadenopathy, chills, and fatigue [28]. Subsequently, a rash appears, typically initiating on the face before spreading to other body regions [7].

The monkeypox rash transitions through various stages: macules, papules, vesicles, pustules, and, ultimately, crusts or scabs [27]. Unlike smallpox, monkeypox typically presents with more pronounced lymphadenopathy (swollen lymph nodes), which may manifest even before the appearance of the rash [20].

Complications

Monkeypox can cause complications, some severe and long-lasting. These include secondary bacterial infections from compromised skin due to pox lesions and life-threatening pneumonia. Sepsis, a systemic reaction to infection, can lead to organ failure and death. Additionally, monkeypox may cause encephalitis, leading to neurological problems or death.

Ocular infections are particularly concerning as they can lead to vision loss. The eyes can become infected either directly by the virus or secondarily by bacteria, leading to complications that can impair vision [29].

Individuals with weakened immune systems are at a heightened risk of developing these complications. For them, monkeypox can be especially severe and can even result in fatal outcomes [22].

Furthermore, even after recovery from the acute phase of the disease, patients are not entirely free from its effects. The pox lesions, which are a hallmark of the disease, can leave behind significant scarring. This scarring can be particularly pronounced if the lesions become superinfected with bacteria, leading to deeper and more extensive tissue damage [15].

Diagnostic techniques

Conventional Diagnostic Methods

Historically, the diagnosis of monkeypox has been predicated on clinical manifestations, patient history, and epidemiological context, including any known exposure to reservoir species or human cases [30]. Laboratory confirmation traditionally involved virus isolation, electron microscopy, or serological assays for orthopoxvirus antibodies. However, these methodologies could be labor-intensive, necessitate specific expertise, and pose a risk of laboratory-acquired infection [31].

Advancements in Diagnostic Techniques

The advent of advanced molecular methodologies has profoundly revolutionized the diagnostic landscape for monkeypox. Techniques such as the polymerase chain reaction (PCR) have emerged as pivotal tools, enabling rapid and precise identification of the disease, even in its nascent stages of infection [32]. Notably, the development and adoption of real-time PCR assays have further enhanced diagnostic precision. These assays are uniquely designed to differentiate the monkeypox virus from other members of the orthopoxvirus family. As a result, they are increasingly being incorporated into both field diagnostic settings and specialized reference laboratories [33].

Furthermore, the integration of next-generation sequencing techniques offers a comprehensive insight into the entire genomic structure of the virus. Such in-depth genomic analyses are invaluable, especially during outbreaks, as they facilitate the accurate tracing of the infection source. Moreover, they provide a deeper understanding of the evolutionary trajectory and genetic variations of the monkeypox virus [34].

Treatment and management strategies

Contemporary Treatment Modalities

The mainstay of treatment for monkeypox primarily encompasses supportive care aimed at symptom alleviation and complication management. This could entail the use of antipyretics to combat fever, analgesics for pain mitigation, and fluids to maintain optimal hydration [35].

Several antiviral drugs, such as cidofovir and its lipid conjugate, brincidofovir, have demonstrated activity against the monkeypox virus in in-vitro conditions and animal models [36]. However, the efficacy of these drugs in humans remains largely unexplored, and they are typically reserved for severe or life-threatening cases due to their potential side effects [37].

For the containment of outbreaks, smallpox vaccine, antiviral drugs, and vaccinia immune globulin can be deployed. The smallpox vaccine has been shown to ameliorate the severity of or even thwart the disease if administered before or shortly after exposure to the virus [38].

Constraints of Existing Treatment Approaches

Despite these available options, the treatment of monkeypox is beset with significant limitations. Firstly, there is an absence of a specific antiviral treatment approved for monkeypox, which implies that care is predominantly supportive [13].

Moreover, although the smallpox vaccine can offer cross-protection against monkeypox, its application is curtailed due to potential side effects and contraindications in specific population groups such as individuals with eczema, pregnant women, and those with compromised immunity [27]. Lastly, the global reservoir of the smallpox vaccine and other potential treatments is finite, which could pose a significant challenge in the face of a large-scale outbreak [7].

Measures for prevention and management

Immunization

The smallpox vaccine, despite the cessation of its routine administration following the eradication of smallpox, has demonstrated effectiveness in offering cross-protection against monkeypox. It continues to be employed in particular scenarios, such as in protecting healthcare workers exposed to the disease at work and during monkeypox outbreaks to curtail further transmission [39].

Despite the potentially severe side effects of the smallpox vaccine, efforts are underway to develop and test a new generation of vaccines that are safer and induce fewer reactions. These could be especially beneficial in managing Monkeypox [40].

Healthcare Policies and Actions

Crucial to the control of monkeypox's spread are public health interventions, which comprise swift detection and isolation of cases, contact tracing, surveillance, and educating the public about risk factors and preventive strategies [35].

In regions where monkeypox is prevalent, attempts should be made to avoid direct interaction with wildlife, particularly with ill or deceased animals, and to ensure all meat is fully cooked before eating [41]. In areas with human cases of the disease, avoiding close physical contact with infected persons and the proper use of personal protective equipment by healthcare workers are significant measures for prevention [42].

Current challenges and controversies

Complications in Disease Management and Prevention

Managing and preventing monkeypox pose several hurdles. Notably, there are restricted resources for monitoring and responding to the disease, especially in remote regions of Central and West Africa, where the virus is most common [22].

The limited comprehension of the carrier species and specific transmission methods of the monkeypox virus in natural settings complicates the development and application of focused preventive measures [43].

Additionally, the discontinuation of smallpox vaccination has resulted in a growing pool of unvaccinated individuals, hence increasing the susceptible population. The administration of the smallpox vaccine for monkeypox prevention is a contentious issue due to its potential adverse effects and the ethical questions raised by using a vaccine meant for a disease that has been eradicated [7].

Challenges in Diagnosis

Correctly diagnosing monkeypox presents significant challenges. Its clinical resemblance to other infectious illnesses, such as chickenpox and even smallpox, can lead to incorrect diagnoses [15].

While molecular techniques have significantly enhanced diagnostic precision, these technologies' limited availability in monkeypox-endemic areas reduces their practicality [20]. Moreover, there is a risk of acquiring the infection in laboratories when dealing with monkeypox samples [32].

Controversies Surrounding Treatment

As of now, no specific antiviral treatment for monkeypox has been approved. The usage of antiviral medications such as cidofovir and brincidofovir is not widespread due to their adverse effects and the absence of large-scale clinical trials proving their effectiveness in humans [36].

Recent developments and future perspectives

Innovations in Vaccine Development

In response to the potential threats posed by monkeypox, substantial progress has been achieved in creating safer, less reactogenic vaccines. One such promising candidate is the modified vaccinia Ankara (MVA). MVA is a considerably attenuated variant of the vaccinia virus that can't replicate in human cells, rendering it safer for people with compromised immune systems. Clinical trials have demonstrated that it elicits robust immune responses against orthopoxviruses, including monkeypox [44].

Potential Antiviral Therapies

Efforts are also ongoing to develop specific antiviral medicines for monkeypox. Tecovirimat is an example of such a development; it's the first medication approved by the FDA for smallpox treatment and has demonstrated activity against the monkeypox virus in laboratory conditions. Clinical trials assessing its effectiveness in treating monkeypox could lay the groundwork for a disease-specific antiviral treatment [45].

Future Avenues for Research

Despite recent progress, many aspects of monkeypox remain mysterious. Future research endeavors should strive to clarify the ecology of the monkeypox virus, specifically its natural hosts and transmission methods. Such insights could guide the creation of more efficient and targeted public health measures [18].

Additionally, investigations are needed to examine the long-term immune responses to new vaccines, the practicality of their wide-scale deployment in regions where the disease is endemic, and strategies to handle potential side effects [46].

Finally, considering the potential of monkeypox to spread worldwide, the disease should be prioritized in One Health initiatives, which acknowledge the interrelation of human, animal, and environmental health [46].

Global health implications

Impact on Public Health

The rising incidence of monkeypox, particularly in areas with low smallpox vaccination rates, highlights its emerging importance as a global public health concern [22]. The disease not only leads to illness and death among affected individuals but also puts a substantial strain on healthcare systems, especially in areas with limited resources [2].

Response Strategies

To address the global menace of monkeypox, a comprehensive and multifaceted response strategy is essential. This encompasses enhancing surveillance systems, improving laboratory diagnostic capabilities, and strengthening infection prevention measures in healthcare facilities [20].

Additionally, planning for preparedness and prompt response to outbreaks are key aspects of this strategy. This approach includes swift identification of cases, isolation, contact tracing, and suitable clinical management [40].

The Role of International Health Organizations

Global health bodies like the World Health Organization (WHO) and the Centers for Disease Control and Prevention (CDC) are critical in steering worldwide efforts to counter monkeypox. They offer technical assistance and advice to countries, carry out disease surveillance, and coordinate international research initiatives [41].

These organizations also assist in formulating prevention strategies, such as immunization programs, and raise awareness about the disease among healthcare providers and the general public [1]. Their role in promoting collaboration among researchers, medical professionals, policymakers, and communities is vital in the global battle against monkeypox.

## Conclusions

Monkeypox has become an increasing concern in the global health landscape. The disease is caused by the monkeypox virus and is primarily found in Central and West Africa, but its capacity for worldwide spread has been demonstrated with cases reported in countries like the United States, the United Kingdom, Israel, and Singapore. The discontinuation of routine smallpox vaccinations has resulted in a growing number of susceptible individuals and a corresponding increase in monkeypox cases.

Despite bearing clinical resemblances to smallpox, monkeypox poses unique difficulties in terms of diagnosis, treatment, and prevention. This highlights the urgency for dedicated research efforts and public health strategies. Notable progress has been made in creating safer vaccines and potential antiviral treatments, yet there is a need for a more detailed examination of their long-term efficacy, potential side effects, and the feasibility of large-scale administration in endemic regions.

Additional investigations are required to better understand the natural reservoirs and transmission pathways of the monkeypox virus. Moreover, research endeavors should be directed toward the creation and evaluation of new vaccines and antiviral treatments. Monkeypox should be a priority in One Health initiatives, which emphasize the interconnected nature of human, animal, and environmental health. In light of the potential for global transmission of monkeypox, international cooperation is paramount. This involves bolstering global and regional disease surveillance systems, the sharing of research data, and coordinating public health responses.
